# Diversity of Rainbow Trout Blood B Cells Revealed by Single Cell RNA Sequencing

**DOI:** 10.3390/biology10060511

**Published:** 2021-06-09

**Authors:** Pedro Perdiguero, Esther Morel, Carolina Tafalla

**Affiliations:** Fish Immunology and Pathology Group, Animal Health Research Center (CISA-INIA), Valdeolmos, 28130 Madrid, Spain; perdiguero.pedro@inia.es (P.P.); morel.esther@inia.es (E.M.)

**Keywords:** teleost, B cells, single cell transcriptomics, immunoglobulins, immune markers, transcription factors, long non-coding RNAs

## Abstract

**Simple Summary:**

Although evolutionarily jawed fish constitute the first group of animals in which a complete adaptive immune system based on immunoglobulins (Igs) is present, many structural immune differences between fish and mammals predict important functional and phenotypical differences between B cells in these two animal groups. However, to date, very few tools are available to study B cell heterogeneity and functionality in fish. Hence, thus far, antibodies targeting the different Igs have been almost exclusively applied as tools to investigate B cell functionality in fish. In the current study, we used the newly developed 10× Genomics single cell RNA sequencing technology and used it to analyze the transcriptional pattern of single B cells from peripheral blood. The results obtained provide us with a transcriptional profile at single cell level of what seem to correspond to different B cell subsets or B cells in different stages of maturation or differentiation. The information provided will not only help us understand the biology of teleost B cells, but also provides us with a repertoire of potential markers that could be used in the future to differentiate trout B cell subsets.

**Abstract:**

Single-cell sequencing technologies capable of providing us with immune information from dozens to thousands of individual cells simultaneously have revolutionized the field of immunology these past years. However, to date, most of these novel technologies have not been broadly applied to non-model organisms such as teleost fish. In this study, we used the 10× Genomics single cell RNA sequencing technology and used it to analyze for the first time in teleost fish the transcriptional pattern of single B cells from peripheral blood. The analysis of the data obtained in rainbow trout revealed ten distinct cell clusters that seem to be associated with different subsets and/or maturation/differentiation stages of circulating B cells. The potential characteristics and functions of these different B cell subpopulations are discussed on the basis of their transcriptomic profile. The results obtained provide us with valuable information to understand the biology of teleost B cells and offer us a repertoire of potential markers that could be used in the future to differentiate trout B cell subsets.

## 1. Introduction

To date, most studies have agreed on the fact that adaptive immunity appeared during the early stages of vertebrate evolution, most likely in the disappeared placoderms [1]. Accordingly, the genes that define the adaptive immune system such as immunoglobulins (Igs), T cell receptors (TCR), major histocompatibility complex I (MHC I), MHC II, recombination activating gene 1 (RAG1), and RAG2 are present in gnathostomes (jawed vertebrates) including cartilaginous fish such as sharks (the most ancient jawed fish) and teleost fish. While the basic components of adaptive immunity are present, it must be taken into account that the adaptive branch of the immune system continued to evolve in tetrapods, reaching further degrees of specialization and sophistication in mammals. Consequently, there are important differences between the mammalian and teleost adaptive immune system that significantly condition the phenotype and functionality of B cells and how they respond to an antigen encounter. For instance, given the lack of bone marrow, the head kidney is the main hematopoietic organ in teleosts, and the main site for B cell differentiation. Similarly, fish do not have lymph nodes, being the spleen the main secondary immune organ. Within the spleen, the organization of lymphocytes is very primitive, with scattered B and T cells and no clearly defined regions as those found in mammals [2,3]. Thus, no cognate germinal centers (GCs) have ever been identified in the teleost spleen. GCs, formed in mammals during the immune response, promote the close collaboration between proliferating antigen-specific B cells, T follicular helper cells, and specialized follicular dendritic cells (DCs). In this environment, B cells divide in response to antigens and acquire the capacity to differentiate into antibody-secreting cells (ASCs), reaching a terminal state of plasma cells or memory B cells, both having the capacity to secrete high affinity antibodies. It is in these sites, that B cells undergo class switch recombination (CSR) and replace the heavy chain of IgM for IgG, IgA or IgE, antibodies with higher affinity and different effector functions. In the absence of GCs or specialized Igs, whether teleost B cells differentiate to plasma cells or memory B cells equivalent to those found in mammals is still a matter of debate. 

Fish also have a more limited Ig array. Fish genomes encode only three classes of Igs, namely IgM, IgD, and the fish specific IgT/Z [4,5]. Thus, in contrast to mammals, no CSR has ever been reported in fish. IgM and IgD are co-expressed on the surface of naïve B cells. In this case, alternative splicing between the recombined variable region and constant regions of IgM and IgD render cells that co-express IgM and IgD of the same specificity. Upon activation, naïve B cells lose surface IgD to become IgM^+^IgD^−^ B cells with a plasmablast profile [6,7]. Moreover, through some still unknown mechanisms, certain cells lose surface IgM and become IgD^+^IgM^−^ B cells. These cells have been identified in catfish blood [8] as well as in rainbow trout gills [9] and gut [10] and have yet unknown functions. Finally, fish B cells expressing IgT cells constitute an independent B cell linage in which IgM and IgD are not expressed [11]. Most teleost species express more than one IgT, three in the case of salmonids [12], although whether individual cells express one or more IgT is still not clear.

To date, antibodies targeting the different Igs have been almost exclusively applied as tools to investigate B cell functionality in fish. In some cases, antibodies recognizing key transcription factors involved in B cell development in mammals (Pax5, Blimp1) have been combined with antibodies recognizing species-specific immunoglobulins (IgM, IgT, and IgD), allowing the identification of B cells in different maturation/differentiation stages by flow cytometry [13,14]. Other studies have focused on identifying in fish orthologue genes of cluster of differentiation (CD) molecules previously characterized and assigned in mammals to specific B cell subsets. However, it should be taken into account that many of these orthologues to key mammalian markers are absent in the teleost genomes or in other cases show a high divergence compared to mammalian proteins, questioning whether they have a similar function. Despite these efforts, the notorious lack of fish-specific markers that differentiate B cell subsets, maturation stages, or differentiated cells have considerably hindered our understanding of teleost B cell functionality. 

The recent development of sophisticated tools that permit the analysis of transcriptomes at single cell resolution is allowing a fast progression of our current knowledge regarding the phenotype and function of different leukocyte subsets. Thus, for example, single cell RNA sequencing has been used in Atlantic cod (*Gadus morhua*) spleen cells and peripheral blood leukocytes, identifying the major cell subsets including cytotoxic T cells, B cells, erythrocytes, thrombocytes, neutrophils, and macrophages [15]. Focusing the analysis on one specific immune cell subset, Niu et al. defined different subsets of non-specific cytotoxic cells in Nile tilapia (*Oreochromis niloticus*) [16]. In the present work, we have taken advantage of the recent single cell genomic tools developed to perform an in depth analysis of teleost B cell transcriptomes at single cell resolution. For this study, we used rainbow trout (*Oncorhynchus mykiss*) as a model species and blood as a B cell source given that this is where the higher percentage of B cells are found in homeostasis [17]. To avoid cross-linking of the B cell receptor (BCR) and the subsequent cell activation in response to anti-IgM, we sorted lymphoid (small cells with low complexity) MHC II^+^ cells, which exclusively corresponded to B cells. Our results evidence the presence of different circulating B cell subpopulations with interesting differences at transcriptional level that seem to reflect different stages of maturation or diverse B cell subsets. These results provide valuable information to elucidate B cell functionality in teleosts. The identification of specific markers for each of these subpopulations will also be of great help to generate in the future novel antibodies that can be used to differentiate teleost B cell subsets.

## 2. Materials and Methods

### 2.1. Experimental Fish

Female rainbow trout (*Oncorhynchus mykiss*) of approximately 50–70 g (15–20 cm) were obtained from *Piscifactoria Cifuentes* (Guadalajara, Spain). Fish were maintained at the Animal Health Research Centre (CISA-INIA) laboratory at 16 °C with a re-circulating water system and 12:12 h light:dark photoperiod. The fish were fed a commercial diet twice a day (T4 Royal Optima, Skretting, Spain). The fish were acclimatized to laboratory conditions for at least 2 weeks prior to any experimental procedure. During this period, no clinical signs were ever observed. The procedures described comply with the Guidelines of the European Union Council (2010/63/EU) for the use of laboratory animals and were previously approved by the Ethics committee from the *Instituto Nacional de Investigación y Tecnología Agraria y Alimentaria* (INIA; Code CEEA PROEX002/17). 

### 2.2. Peripheral Blood Leukocyte Isolation and Sorting 

Three individual rainbow trout were killed by benzocaine (Sigma) overdose. Blood was extracted with a heparinized needle from the caudal vein and diluted 10 times with Leibovitz medium (L-15, Thermo Fisher Scientific, Walthan, MA, USA) supplemented with 100 IU/mL penicillin and 100 μg/mL streptomycin (P/S, Thermo Fisher Scientific), 2% fetal calf serum (FCS, Thermo Fisher Scientific), and 10 IU/mL heparin (Sigma, St. Louis, MO, USA). Peripheral blood leukocytes (PBLs) were obtained by density gradient centrifugation (500× *g* for 30 min at 4 °C) of diluted blood on 51% continuous Percoll (GE Healthcare, North Richland Hills, TX, USA). The interface cells were collected, washed twice in L-15 containing antibiotics and 5% FCS and adjusted to 2 × 10^6^ cells/mL.

To avoid the activation of B cells by BCR cross-linking and to be able to study different B cell subsets independently of their pattern of surface Ig expression, we decided to sort MHC II^+^ cells with small size and low complexity (within what has been previously designated as the lymphoid gate), as it was predicted that these cells would correspond mainly to B cells. For this, PBLs were washed in FACS staining buffer (phenol red-free L-15 medium supplemented with P/S and 2% FCS) and incubated with a monoclonal antibody specific for rainbow trout MHC II β chain (mAb mouse IgG1 coupled to allophycocyanin, 2 μg/mL) previously characterized [18]. After 30 min of incubation at 4 °C, the cells were washed with FACS staining buffer and YO-PRO dye (0.05 μM) added to the cell suspension for dead cell exclusion. Lymphoid (small, low complexity) MHC IIβ^+^ YO-PRO^−^ (live) cells were then isolated in a FACSAria™ III sorter (BD Biosciences, San Jose, CA, USA) equipped with BD FACSDiva™ software (BD Biosciences). The purity of the sorted population (above 98%) was confirmed in a FACS Celesta flow cytometer (BD Biosciences).

### 2.3. Library Construction and Sequencing

Isolated MHC II^+^ cells gently pipetted and diluted to a concentration of 700 cells/µL were used for cell isolation on a 10× Genomics Chromium Controller instrument [19]. All steps, including PBL isolation, sorting, and Chromium™ Single Cell isolation were carried out in the same morning to avoid cell death. A total of 2500 cells per donor were loaded into the chips of the Chromium™ Single Cell 5′ Gel Beads Kit (10× Genomics) and subjected to the Chromium Controller instrument to generate single cell Gel Bead-In Emulsions (GEMs) following the manufacturer’s instructions. Next, GEMs were subjected to library construction using the Chromium™ Single Cell 5′ Library Kit v1 (10× Genomics). As a first step, reverse transcription was performed, resulting in cDNA tagged with a cell-specific barcode and a unique molecular index (UMI) per transcript. Fragments were then size selected using SPRIselect magnetic beads (Beckman Coulter). Next, Illumina sequencing adapters were ligated to the size-selected fragments and cleaned up using SPRIselect magnetic beads (Beckman Coulter, Brea, CA, USA). Finally, sample indexes were incorporated and amplified, followed by a double-sided size selection using SPRIselect magnetic beads (Beckman Coulter). The quality of the final library was assessed using an Agilent 2100 Bioanalyzer (Agilent technologies, Amstelveen, The Netherlands). The samples were then sequenced using a NextSeq instrument (Illumina) with 150 PE chemistry. 

### 2.4. Alignment and Initial Processing of the Sequencing Data

The Cell Ranger software (10× Genomics, v3.1) was used to process the sequenced libraries. A rainbow trout reference transcriptome was constructed from the RefSeq *Oncorhynchus mykiss* genome v1.0 using the “Cell Ranger mkref” tool. The sequences encoding the constant regions of rainbow trout Igs were added to the fasta file prior to the construction of the reference transcriptome. The complementary DNA reads from each donor were mapped against this reference using the “Cell Ranger count” tool. Through this system, filtered UMI expression matrices from each donor were generated. As a result, the raw expression data was obtained containing transcriptomes for single MHC II^+^ cells from the three donor fish.

In accordance with published pipelines and quality control standards, abnormal cells in all datasets were uniformly filtered out based on their gene expression distribution using the Seurat package (version 3.1) [20,21]. Cells with at least 200 detected genes, and only those genes that appeared at least in three cells were included in the initial matrix from each fish. A cell was considered to be abnormal if any of the following criteria were met: (i) detected gene number >2500; (ii) detected count number >15,000 and (iii) >25% of reads mapping for mitochondrial genes or no reads mapping for mitochondrial genes.

### 2.5. Data Integration and Cell Clustering

The SCTransform method from the Seurat software was applied in order to normalize the three filtered single-cell datasets from different fish. The percentages of mitochondrial and ribosomal proteins previously calculated for each cell were included as variables to regress the data. Then, filtered and normalized datasets were integrated using the PrepSCTIntegration tool to avoid a batch effect, enabling a systematic comparison between the three fish. The merged data was subjected to dimensionality reduction using principal component analysis (PCA) followed by uniform manifold approximation and projection (UMAP) using the first 30 principal components (PCs). This setup was also used to define nearest neighbors among cells with the KNN method using the FindNeighbors function. To group the cells in different subsets according to expression levels, the FindCluster tool was applied using the Louvain algorithm with the resolution set as 0.5, which allowed the correct definition of clearly separated clusters.

### 2.6. Marker Identification and Functional Analysis

The identification of genes showing differential expression associated to a specific cluster was performed using the FindAllMarkers tool from the Seurat software, considering a significant association for those genes showing an adjusted *p* < 0.001 and logFC ≥ 0.25. The information relative to the gene description contained in the *O. mykiss* genome v1.0 was taken into account for gene name association. In order to obtain an actualized functional annotation, the nucleotide sequences from the genes identified as markers were compared with proteins from a set of model species (*Homo sapiens*, *Mus musculus*, *Danio rerio*, *Macata mulata*, *Drosophila melanogaster*, and *Xenopus tropicalis*) using the Blastx software applying as threshold a minimum E value of 10^−5^. The blast results were subjected to the Blast2GO software for GO term mapping. Sequences were also compared against domain databases using the InterProScan tool implemented in Blast2GO. GO term annotations were inferred for rainbow trout transcripts. Single enrichment analysis was performed by comparing the functions associated to genes from each cluster taking into account differences with an adjusted *p* < 0.05. 

## 3. Results

### 3.1. Single-Cell Transcriptomic Analysis of Rainbow Trout MHC II^+^ Lymphoid Cells from Peripheral Blood

To acquire a transcriptomic profile of rainbow trout peripheral blood B cells at single-cell resolution, leucocyte isolation following sorting of lymphoid (small size and low complexity) MHC II^+^ cells was carried out using blood obtained from three independent unstimulated fish. Cell viability was checked after sorting and confirmed to be higher than 90% in all samples. Single cell cDNA libraries were sequenced using Illumina HiSeq 150PE obtaining a total number of 73,934,335, 83,626,235 and 76,465,953 raw reads per fish. After quality control and mapping using the Cell Ranger software, transcriptomes of 1078, 2178, and 1488 single cells from each fish were acquired, detecting a median of 762, 694, and 1027 genes per cell, respectively. 

**Figure 1:**
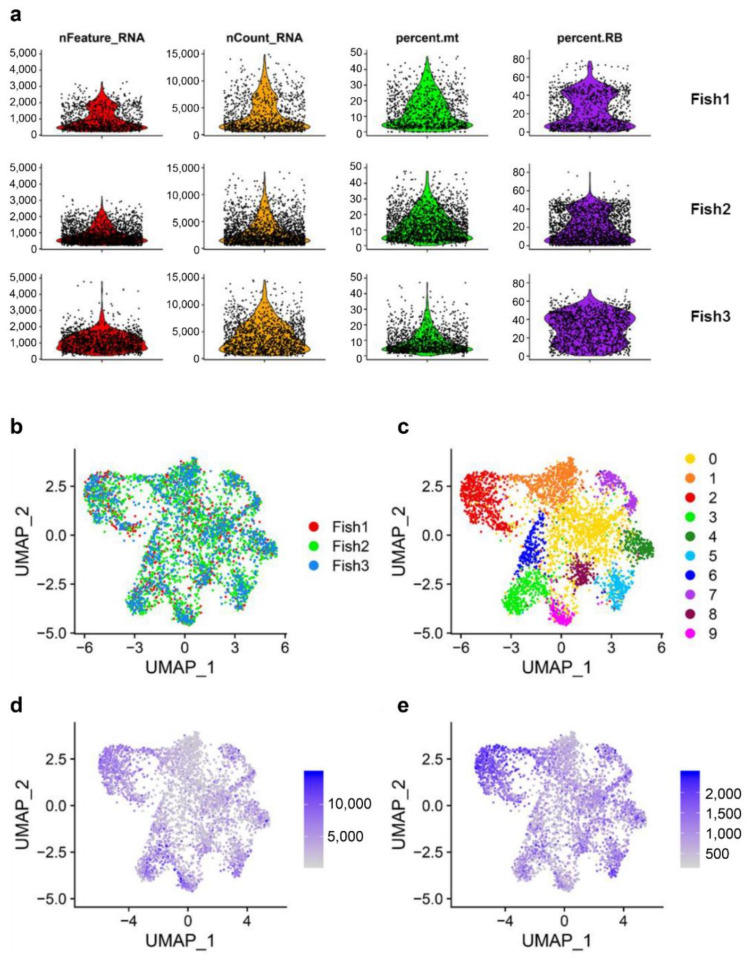
Single cell sequencing quality check and cell projection analysis. (**a**) Quality check of single cell sequencing results. The figure shows the total number of genes (nFeature_RNA), the total number of reads (nCount_RNA), the percentage of reads mapping mitochondrial genes (percent.mt) and the percentage of reads mapping ribosomal proteins (percent.RB) per cell in each individual fish. UMAP visualization of cells integrating data from the three individual fish (**b**); defining clusters of cells with similar transcriptional identities (**c**); showing the total number of reads identified for each cell (**d**) or the total number of genes identified for each cell (**e**).

Single cell sequencing data from each fish was then analyzed to determine the distribution of genes, read counts, percentage of reads mapping mitochondrial genes as well as percentage of reads mapping ribosomal proteins (Figure 1a). The distribution of genes and reads was found to be similar in all fish, with approximately 2500 genes per cell or 15,000 counts per cell (Figure 1a). Some cells, mainly identified in Fish 3, showed a higher number of genes or reads. These cells were considered abnormal cells or potential doublets and were filtered out for successive analysis. Regarding the percentage of mitochondrial reads, a wide distribution was observed in all fish reaching values of ~50% (Figure 1a). Commonly, a high percentage of mitochondrial reads is associated with abnormal cells or cells that have been damaged during isolation. For this reason, cells showing a percentage of mitochondrial reads higher than 25% were considered abnormal and excluded from subsequent analysis. Similarly, a reduced number of cells with no mitochondrial reads were filtered out at this step. After filtering a total of 843, 1814, and 1327 cells were retained for successive analysis.

Once the data was normalized and integrated, the cells from each of the three fish appeared to be equally distributed along the cell projection (Figure 1b). After cell clustering, a global view was generated to illustrate the potential subpopulations of rainbow trout peripheral blood MHC II^+^ B cells. It should be noted that 99.28% of the cells analyzed transcribed some type of B cell marker, either genes encoding Ig heavy or light chains or isoforms of CD79, previously identified as a B cell specific marker in Atlantic salmon (*Salmo salar*) [22], confirming that almost all MHC II^+^ lymphoid cells in peripheral blood correspond to B cells. The UMAP reduction generates a clear cell clustering highlighting 10 distinct cell populations based on their gene expression profiles (Figure 1c). All the clusters identified were shared by the three fish analyzed (Figure 1b). According to the global expression analysis, the clusters located at the central region of the cell projection (Clusters 0, 1, and 6) seem to contain cells showing a lower number of reads (Figure 1d) as well as a lower number of expressed genes per cell (Figure 1e). In contrast, the clusters located in the periphery of the cell projection show cells with higher number of reads and genes expressed, especially noticeable for clusters 2, 4, 5, and 7 (Figure 1d,e).

### 3.2. Characterization of Marker Transcripts Associated with Identified Cell Clusters

**Figure 2:**
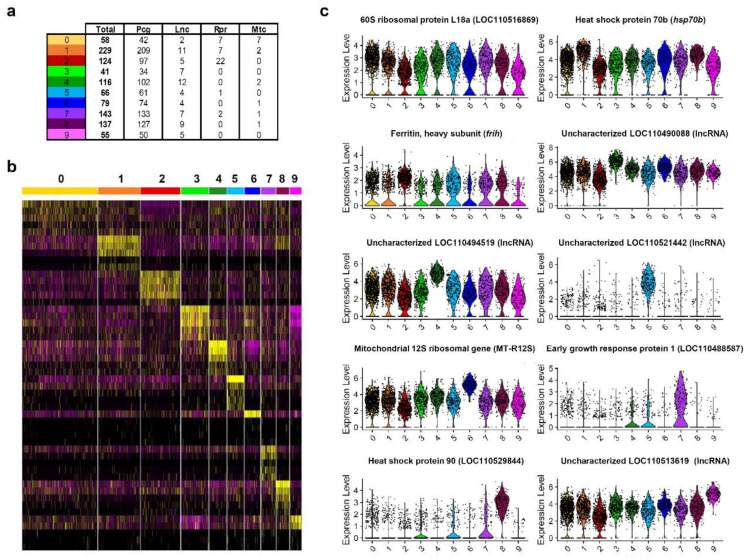
Identification of transcripts specific for each rainbow trout B cell subset. (**a**) Table including the number of transcripts identified for each cell cluster showing significant differences in expression levels (adjusted to *p* < 0.001) differentiating among protein coding genes (Pcg), long non-coding RNAs (Lnc), genes coding for ribosomal proteins (Rpr) and mitochondrial genes (Mtc). (**b**) Heatmap showing expression levels from the top five most significant subset-specific transcripts for each B cell subset (**c**) Violin plots showing normalized expression levels in all B cell subsets for the most significant marker transcript in each B cell subset.

Following the identification of cell clusters of B cells according to their transcriptional profile, a new analysis was performed to identify those genes that were most specific to a given cluster versus the rest. These specific marker transcripts identified for each cluster were retained when the adjusted *p* value was lower than 0.001 (Table S1). These transcripts that showed significant differences in transcription levels among different B cell subsets, included protein-coding genes as well as genes encoding ribosomal proteins, long non-coding RNAs (lncRNAs), and also mitochondrial genes (Figure 2a,b). Specifically, the gene encoding the 60S ribosomal protein L18a (LOC110516869) was the gene that most significantly represented cluster 0 (Figure 2c). Genes encoding the heat shock protein 70b (hsp70b), the ferritin heavy subunit (frih), an early growth response protein 1 (LOC110488587), and the heat shock protein HSP 90-alpha-like protein (LOC110529844) were the genes that most significantly differentiated clusters 1, 2, 7, and 8 respectively (Figure 2c). Interestingly, clusters 3, 4, 5, and 9 had one lncRNA as their most significant marker transcript (Figure 2c). Finally, cells included in cluster 6 showed a remarkable expression of the mitochondrial 12S ribosomal gene (Figure 2c). 

#### 3.2.1. Description of Molecular Markers Associated with Different B Cell Subsets 

Marker transcripts for each B cell subset as well as the potential functions associated to each of these genes were further analyzed using the GO term single enrichment analysis. Several of these genes for which significant differences in expression levels were identified among the different clusters included genes encoding important enzymes, genes related to immune functions, as well as several transcription factors involved in important physiological processes (Table S1). Interesting functionalities were also associated with each B cell subset through this analysis (Table S2). The most relevant findings are summarized below. 

##### Cluster 0

**Figure 3:**
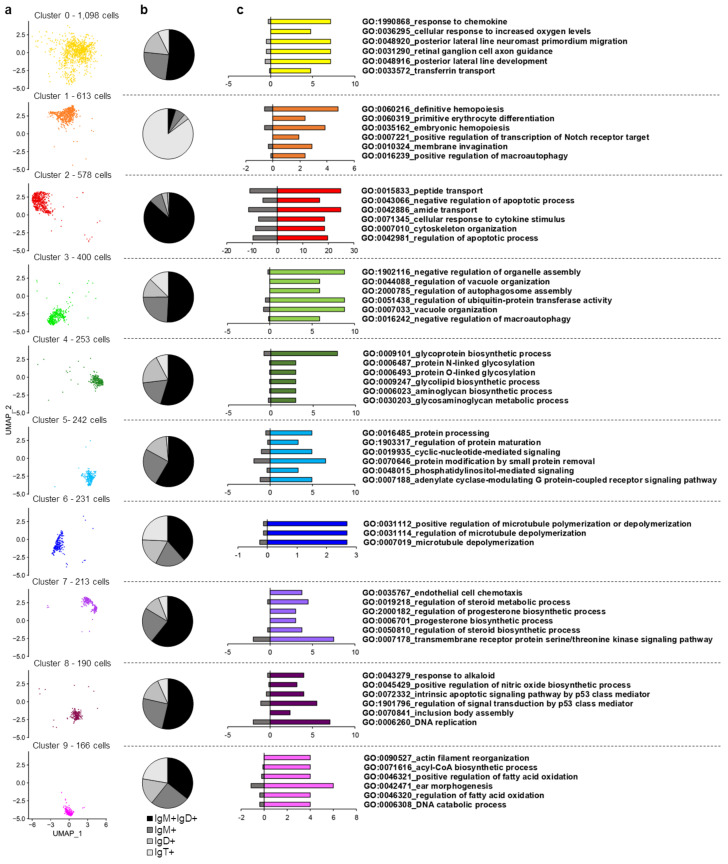
Cluster definition including immunoglobulin profile of cells and functional analysis of marker transcripts. (**a**) UMAP visualization of cells in each cluster. (**b**) Percentage of cells within each cluster assigned to common heavy chain Ig subsets (IgM^+^IgD^+^, IgM^+^, IgD^+^ and IgT^+^) according to their transcriptional analysis. (**c**) Single enrichment analysis along the different cell clusters. Graphs show the percentage of genes within the most significant GO terms at level six within the biological process category for each specific cluster (colored bars) versus the percentage of genes from this category found in the rest of clusters (grey bars).

**Figure 4:**
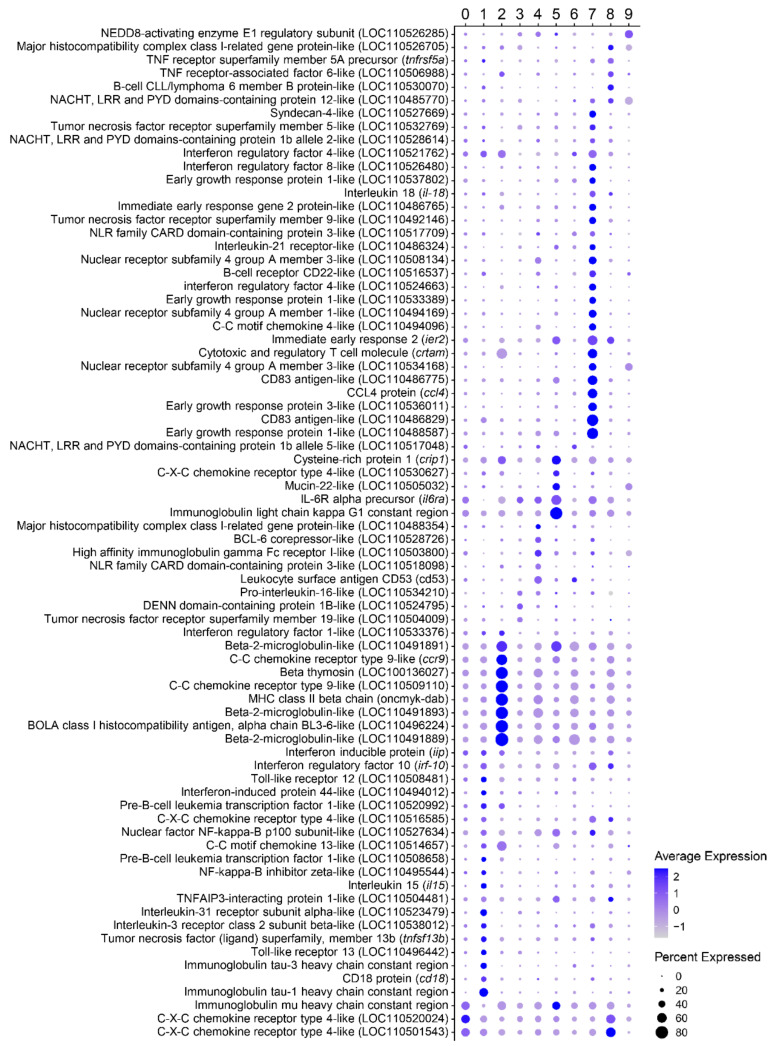
Dot plot analysis showing the marker transcripts identified for each cell cluster related with immune function. For each gene, the average normalized expression (dot color) together with the percentage of cells expressing the gene (dot size) are shown for each cluster.

Cluster 0 is the largest cluster in number of cells, with 1098 cells in total (taking into account all three fish), and it is located at the center of the cell projection (Figure 3a). According to the transcription of the constant regions of Ig genes around 50% of cells within this cluster are IgM^+^IgD^+^ B cells (Figure 3b). A significant enrichment of GO terms “response to chemokines” (GO:1990868) or “cellular response to increased oxygen levels” (GO:0036295) was observed in this cluster within the biological process category (Figure 3c). A gene encoding an interferon-induced protein with tetratricopeptide repeats 5-like (LOC110491392) or a transmembrane protein 42-like (LOC110516550) were found within the genes showing the highest differences in expression levels for this cluster (Table S1). Some interesting immune genes were also found transcribed at significantly higher levels in this cluster. These included genes coding for the constant region of the immunoglobulin mu heavy chain and a C-X-C chemokine receptor type 4-like (LOC110520024 and LOC110501543), which also showed higher expression in Cluster 8 (Figure 4).

##### Cluster 1

**Figure 5:**
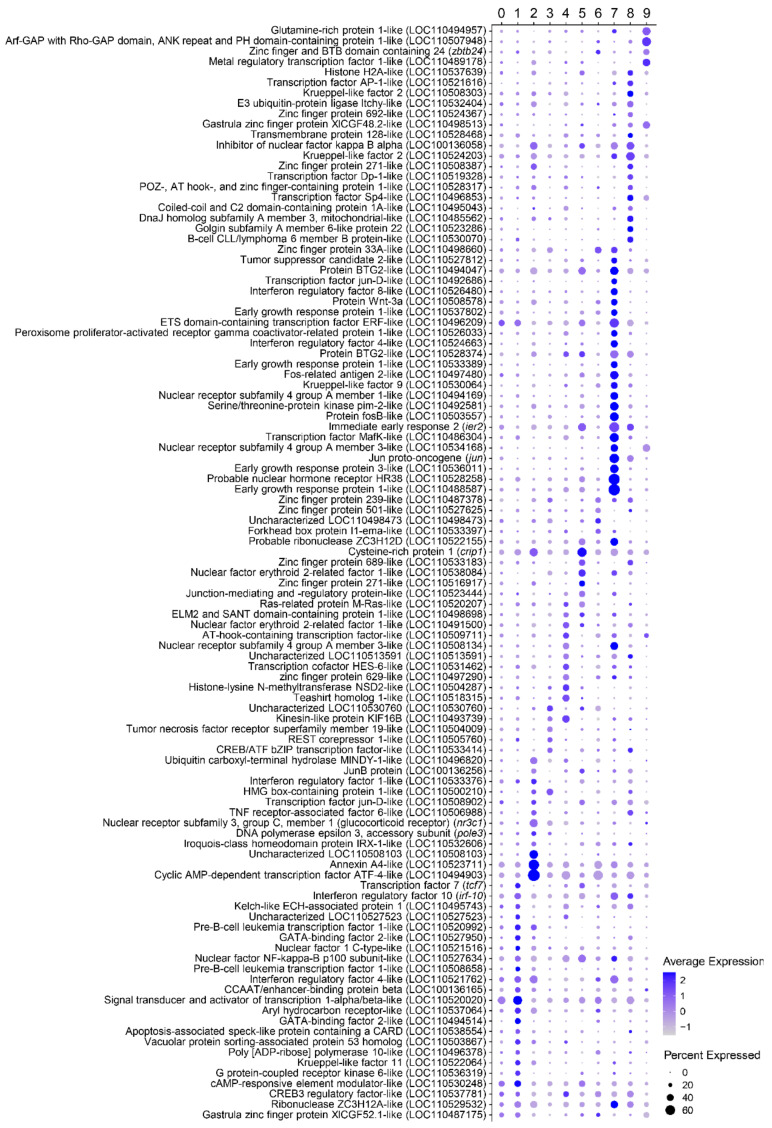
Dot plot analysis including those marker transcripts identified for each cell cluster related to the activity of transcription factors. For each gene, the average normalized expression (dot color) together with the percentage of cells expressing the gene (dot size) are shown for each cluster.

Cluster 1 in formed by 613 cells and is located in the upper region of cell projection (Figure 3a). This B cell subset is mainly represented by IgT^+^ B cells (Figure 3b). Intriguingly, this cluster is characterized by a group of markers related with “definitive hemopoiesis” (GO:0060216), “primitive erythrocyte differentiation” (GO:0060319) and “positive regulation of transcription of Notch receptor target” (GO:0007221) (Figure 3c). Regarding gene expression, this cluster is mainly represented by cells expressing immunoglobulin tau-1 and tau-3 heavy chains. Several genes coding for homologues of heat shock protein 70 also appeared among the most significant markers associated with this cluster (*hsp70b*, LOC110486254, LOC110485160, and LOC110486256) (Supplementary Table S1). Other immune genes transcribed at significantly higher levels by several cells within this cluster include genes coding for CD18 (*cd18*), interleukin 15 (*il15*), interleukin 31 receptor subunit alpha-like (LOC110523479), interleukin 3 receptor class 2 subunit beta-like (LOC110538012), tumor necrosis factor superfamily member 13b (*tnfsf13b*), as well as a gene encoding a CXC chemokine receptor type 4-like (LOC110516585) (Figure 4). Some receptors related to innate immune responses were also highly transcribed in cells from this cluster, for example, toll-like receptor 12 (LOC110508481) or 13 (LOC110496442) (Table S1). Among genes that encode transcription factors, these cells are characterized by the transcription of two genes encoding GATA-binding factors 2-like proteins (LOC110494514 and LOC110527950), involved in primitive hematopoiesis in mammals. As mentioned above, some genes related with regulation of the Notch pathway were also up-regulated in this cell cluster. These included two genes encoding pre-B-cell leukemia transcription factors 1-like proteins (LOC110508658 and LOC110520992), and a gene encoding a signal transducer and activator of transcription 1-alpha/beta-like protein (LOC110520020) (Figure 5). Analyzing the GO terms within the molecular function category associated with this cluster, we found “signaling receptor binding” (GO:0005102) and “calcium ion binding” (GO:0005509) (Table S2). In correlation, a representative group of genes highly expressed in cells from this cluster are related with calcium regulation, for example genes encoding ictacalcin (*s10i*), an ictacalcin-like protein (LOC110520890), a C-type lectin domain containing 14A (*clec14a*), and a E3 ubiquitin-protein ligase CBL-like protein (LOC110533571) (Table S1). 

##### Cluster 2

Cluster 2, located in the left part of the cell projection, is formed by a total of 578 cells (Figure 3a). The cluster is mostly composed of IgM^+^IgD^+^ B cells, which constitute approximately 87% of these cells according to the levels of transcription of Ig constant regions (Figure 3b). Cluster 2 seems to be the most transcriptionally active cell cluster according to number of reads and genes transcribed per cell (Figure 1e). Genes identified as marker genes specific for this cluster are related to several functions including “peptide transport” (GO:0015833), “negative regulation of apoptotic process” (GO:0043066), “cellular response to cytokine stimulus” (GO:0071345), or “positive regulation of protein binding” (GO:0032092) (Figure 3c, Table S2), among others functionalities (Table S2). Among the genes most significantly expressed by this cluster we found some interesting immune genes, such as two genes encoding CC chemokine receptor type 9 (*ccr9* and LOC110509110), different components of the MHC II complex, including the MHC class II beta chain (*oncmyk-dab*) and the BOLA class I histocompatibility antigen, an alpha chain BL3-6-like (LOC110496224), and several homologues of beta-2-microglobulins (LOC110491889, LOC110491891 and LOC110491893) (Figure 4). In addition, among genes associated with the activity of transcription factors, we found a strong transcription of the factor BTF3 (LOC110523362), annexin A4-like (LOC110523711) and the cyclic AMP-dependent transcription factor ATF-4 (LOC110494903) (Figure 5). Finally, beta thymosin (LOC100136027), a gene associated with plasma cell differentiation, was also identified among the most significant marker transcripts for this cluster (Table S1). 

##### Cluster 3

A total of 400 cells were identified in cluster 3, located at the bottom left region of the cell projection (Figure 3a). According to the levels of transcription of Ig constant regions, this cluster included IgM^+^IgD^+^ cells, but also IgT^+^, IgD^+^ or IgM^+^ cells (Figure 3b). The numbers of marker transcripts identified for this cluster were lower than for other clusters but were enriched in functions such as “negative regulation of organelle assembly” (GO:1902116), “regulation of ubiquitin-protein transferase activity” (GO:0051438) and “regulation of gene expression, epigenetic” (GO:0040029) (Figure 3c, Table S2). A gene coding for the PTEN induced putative kinase 1 (*pink1*) was transcribed at significantly higher levels in cells from this cluster, despite the fact that this gene was already highly transcribed in all the cells analyzed. A gene encoding a lysosomal acid phosphatase-like protein (LOC110506315) was also preferentially transcribed in this cluster (Table S1). Concerning immune genes, some cells from this cluster highly expressed a gene coding for a tumor necrosis factor receptor superfamily member 19-like protein (LOC110504009) and a DENN domain-containing 1B-like protein (LOC110524795) (Figure 4). Additionally, a few genes related to transcription factor activity were identified with higher expression in this cluster, such as genes coding for a CREB/ATF bZIP transcription factor-like protein (LOC110533414) and a REST corepressor 1-like protein (LOC110505760) (Figure 5).

##### Cluster 4

A group of 253 cells localized at the right region of cell projection was designated as cluster 4 (Figure 3a). According to the levels of transcription of Ig constant regions, although the majority of cells from this cluster were IgM^+^IgD^+^ cells, IgT^+^, IgD^+^, or IgM^+^ cells were also included (Figure 3b). Interestingly, marker transcripts for this cluster indicate a significant enrichment in functions related to “glycoprotein biosynthetic process” (GO:0009101) as well as “protein N-“ or “O-linked glycosylation” (GO:0006487 and GO:0006493, respectively) (Figure 3c). Among the genes related to these functions that were transcribed at significantly higher levels in this cluster, we found genes coding for several enzymes, such as for example beta-1,3-galactosyltransferase 6-like protein (LOC110527712), beta-1,4-glucuronyltransferase 1 (*b4gat1*), probable C-mannosyltransferase DPY19L3 (LOC110506384), and UDP-GlcNAc:betaGal beta-1,3-N-acetylglucosaminyltransferase 9-like protein (LOC110494808) (Table S1).

##### Cluster 5

A group of 242 cells localized at the bottom right section of the cell projection was annotated as cluster 5 (Figure 3a). The cells from this cluster were mostly IgM^+^IgD^+^ cells but cells transcribing only IgT or IgM were also identified, according to the levels of transcription of Ig constant regions (Figure 3b). The genes identified in this cluster are enriched in functions such as “protein processing” (GO:0016485) or “regulation of inflammatory response” (GO:0050727). (Figure 3c, Table S2). Interestingly, the cells included in this cluster exhibited higher transcription levels of the constant region of immunoglobulin light chain kappa G1. Other genes related with immune functions identified with higher transcription levels in cells from this cluster, included, for instance, genes coding for the IL-6R alpha precursor (*il6ra*), CXC chemokine receptor type 4-like (LOC110530627), mucin-22-like (LOC110505032), or cysteine-rich protein 1 (*crip1*) (Figure 4). Within transcription factors, different zinc finger proteins (585-LOC110515698, 271-LOC110516917, and 689-LOC110533183) were identified with higher expression than other clusters as well as a nuclear factor erythroid 2-related factor 1-like (LOC110538084) (Figure 5).

##### Cluster 6

A total of 231 cells were included in Cluster 6, situated at the left part of cell projection and near cluster 0 (Figure 3a). Cells from this cluster were identified as a mixture of IgM^+^IgD^+^ cells and cells exclusively expressing IgT, IgM or IgD (Figure 3b). Transcripts in this cluster were enriched in a lower number of functionalities than those of other clusters, identifying genes related to “melanocyte differentiation” (GO:0030318) as well as “positive regulation of microtubule polymerization or depolymerization” (GO:0031112) (Figure 3c, Table S2). Cluster 6 is mainly characterized by a high expression of the ribosomal 12S gene mitochondrial gene (Figure 2c). Interestingly, no nuclear genes are clearly associated with cells included in this cluster. 

##### Cluster 7

Cluster 7 is represented by 213 cells located at the upper and right part of the cell projection (Figure 3a). This cluster is mostly composed of IgM^+^IgD^+^ B cells, which constitute approximately 63% of these cells according to the levels of transcription of Ig constant regions (Figure 3b). Functions related with “regulation of steroid metabolic process” (GO:0019218) or “transmembrane receptor protein serine/threonine kinase signaling pathway” (GO:0007178) were found within the most significant functions from the biological process category (Figure 3c). This cluster contains several genes associated to important immune system functions, such as “regulation of cell population proliferation” (GO:0042127), “positive regulation of cytokine production” (GO:0001819), “lymphocyte activation” (GO:0046649), and “cell migration” (GO:0016477) (Table S2). Also, significant enrichments in other GO terms from the molecular function category, such as “signaling receptor activator activity” (GO:0030546) or “DNA-binding transcription activator activity” (GO:0001216) were also detected (Table S2). The analysis of cluster 7 highlighted interesting markers related to immune functions. Thus, genes encoding two CD83 antigen-like proteins (LOC110486829 and LOC110486775), CCL4 protein (*ccl4*), three early growth response protein 1-like (LOC110488587, LOC110533389, and LOC110537802) and other early growth response protein 3-like (LOC110536011), the immediate early response 2 protein (*ier2*), as well as several nuclear receptor subfamily 4 genes (LOC110534168, LOC110508134, and LOC110494169) were identified within the most significant markers for this cluster (Figure 4). Additional immune related genes were expressed in several cells from this cluster, for instance, a C-C motif chemokine 4-like protein (LOC110494096), two genes encoding interferon regulatory factor 4-like proteins (LOC110524663 and LOC110521762), an interferon regulatory factor 8-like protein (LOC110526480), a tumor necrosis factor receptor superfamily member 9-like protein (LOC110492146) and a cytotoxic and regulatory T cell molecule (*crtam*) (Figure 4). 

##### Cluster 8

A total of 190 cells were grouped in cluster 8, located at the center of the cell projection near cluster 0 (Figure 3a). According to the levels of transcription of Ig constant regions, although the majority of cells from this cluster were IgM^+^IgD^+^ cells, IgT^+^, IgD^+^, or IgM^+^ cells were also present in this cluster (Figure 3b). Genes with higher transcription levels in cluster 8 than in other clusters appeared associated to “cellular response to stress” (GO:0033554) and “programmed cell death” (GO:0012501) (Table S2). Within the molecular function category, a significant enrichment in genes identified in the GO terms “heat shock protein binding” (GO:0031072) and “ubiquitin-like protein ligase binding” (GO:0044389) were also detected (Table S2). These enrichments were reflected in the genes that were identified within the most significant marker transcripts for this cluster, which included genes encoding several heat shock proteins as well as several ubiquitins. Among the heat shock proteins, a gene coding for a HSP90-alpha-like protein (LOC110529844) was the most significant marker showing high transcription levels in all cells included in this cluster (Figure 2c). In addition, a group of genes coding for homologues of the HSP70 kDa protein (LOC110485160, LOC110533353 and LOC110486254) also showed remarkable transcription levels in cells from this cluster. Within ubiquitins, genes encoding polyubiquitin-B (LOC110533627), polyubiquitin (LOC110533628), ubiquitin B (*ubb*), or E3 ubiquitin-protein ligase RNF220-like (LOC110508723) also showed significantly higher mRNA levels in cells from this cluster when compared to cells from other clusters (Table S1). Some genes related to immune functions were also identified as marker transcripts for cluster 8, such as genes coding for a B-cell CLL/lymphoma 6 member B-like protein (LOC110530070), a TNF receptor superfamily member 5A precursor (*tnfrsf5a*), and a TNF receptor-associated factor 6-like protein (LOC110506988) (Figure 4). 

##### Cluster 9

A total of 166 cells were grouped in cluster 9, located at the bottom of the cell projection (Figure 3a). Cells in this cluster corresponded to either IgM^+^IgD^+^, IgT^+^, IgM^+^ or IgD^+^ B cells according to their transcriptional Ig profile (Figure 3b). The marker transcripts identified in this subpopulation were mainly associated with functions related to “lipid storage” and “post-translational protein modification” (GO:0043687) (Table S2). This cluster was mainly characterized by the transcription of a gene encoding a glycine-rich RNA-binding -like protein (LOC110508373) (Table S1). Among immune genes, cells from this cluster exhibited higher transcription levels of a cullin-associated NEDD8-dissociated protein 1 (LOC110513018) (Figure 4). Some transcription factors such as the zinc finger and BTB domain containing 24 protein (*zbtb24*) or the metal regulatory transcription factor 1-like protein (LOC110489178) were also transcribed at significantly higher levels by cells in this cluster (Figure 5).

#### 3.2.2. Long Non-Coding RNAs Associated to B Cell Subsets

**Figure 6:**
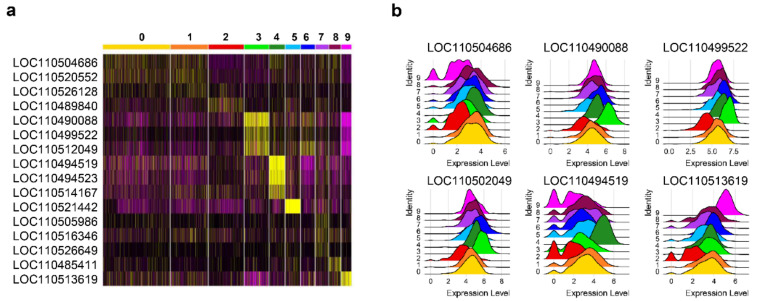
Long non-coding RNAs (lncRNAs) identified as markers for different cell clusters. (**a**) Heatmap showing the most significant lncRNAs with differential expression between clusters. (**b**) Ridge plot showing normalized expression in each cluster of lncRNAs identified as markers for specific subsets.

Several lncRNAs showed differential transcription levels along the different B cell clusters identified among trout peripheral blood B cells (Figure 6a). Remarkably, some of these lncRNAs were found to be widely and strongly expressed all B cells (Figure 6b). 

The lncRNAs LOC110490088, LOC110494519, LOC110521442, and LOC110513619 were identified as the most significant marker transcripts for clusters 3, 4, 5, and 9, respectively (Figure 2c). LOC110504686, LOC110520552, and LOC110495721 were also expressed at slightly higher levels in cells from clusters 0, 1 and 2, respectively (Figure 6a,b). In clusters 3 and 4, in addition to the LOC110490088 and LOC110494519 lncRNAs previously mentioned, several other lncRNAs showed strong expression levels. Thus, LOC110499522, LOC110512049, LOC110510226, and LOC110535625 showed higher transcription levels in cells from cluster 3 whereas LOC110494523, LOC110514167, LOC110514392, and LOC110538866 showed higher transcription levels in cluster 4 (Figure 6a,b). 

## 4. Discussion

Immune studies in mammalian models, especially those undertaken in human and mouse, have identified specific surface markers and/or transcription factors that have allowed the classification of B cells into different subsets and/or different stages of maturation/differentiation. Interestingly, remarkable differences have been observed between human and mouse markers throughout the differentiation process, often sharing just two or three markers between the two species within a specific B cell state [23,24]. In addition, several key markers that define B cell subsets in mammals such as CD19, CD23, or CD24 have no orthologues within the fish genomes, evidencing important differences in B cell biology between mammals and fish. Hence, the analysis of rainbow trout genes encoding homologues to different CD previously described along different states of human and mouse B cells evidenced no expression or residual expression of most of these markers in rainbow trout B cells, with the exception of the different genes encoding CXCR4-like molecules. Surprisingly, CXCR4 was the only marker gene identified in rainbow trout B cells, which can also be considered a marker gene for specific B cell subsets in humans. Other genes identified in our studies as marker genes, have not been identified as such in mammals. Instead, many of these genes correspond to proteins identified in a study recently performed by Peñaranda and collaborators in which a surface proteome of salmon (*Salmo salar*) IgM^+^ B cell was performed [22]. These observations evidence the great differences between markers of B cell subsets between fish and mammals, highlighting the need for high throughput studies that perform an unbiased search for these proteins. Of course, studies such as the one performed here, are not only useful to render a panel of molecules that could be used as markers for specific B cell populations, but also provide further insights on the functionality of teleost B cells which are further discussed. 

### 4.1. Cluster 2 May Represent a Subpopulation of Mature B Cells with High Antigen Presenting Capacities

Cluster 2 was the subpopulation expressing the highest number of genes per cell. This cluster was clearly enriched in IgM^+^IgD^+^ B cells (~86%). In addition, this cluster transcribed several components of the MHC II complex and associated microglobulin genes as the most significant marker genes. A significant enrichment in genes involved in peptide or amide transport, also seems to indicate that this B cell subpopulation is associated with a high antigen presenting capacity. Two genes encoding orthologues of mammalian CCR9 are also interesting markers for this subpopulation. In mammals, CCR9 has been shown to regulate naïve B cell migration [25] and localization of plasma cells in mucosal tissues [26]. In addition, high CCR9 expression has also been revealed in mammalian memory B cells [27]. To date, the function and ligands of fish CCR9 are still unknown. 

Our results also identified a group of transcription factors among the most significant markers of this subpopulation. These include BTF3 as well as the cyclic AMP-dependent transcription factor ATF4. Studies in mammals suggest these transcription factors play different roles associated with the endoplasmic reticulum biology. Thus, BTF3 transcription factor in association with nascent polypeptide-associated complex subunit alpha (LOC110492373), also identified as a marker for this cell cluster, form the nascent polypeptide-associated complex (NAC) which prevents the inappropriate targeting of non-secretory polypeptides to the endoplasmic reticulum. ATF4, on the other hand, has been related with the activation of genes during endoplasmic reticulum stress, a process that regulates activation, B cell differentiation to plasma cell, and cytokine expression mediated by unfolded protein response (UPR) [28]. 

### 4.2. Cluster 7 May Represent a Subpopulation of Activated B Cells with Innate Properties

Teleost B cells retain several innate functions such as a strong phagocytic capacity, ability to produce pro-inflammatory cytokines or antimicrobial peptides in response to a non-specific stimulation [29,30,31]. However, whether all teleost B cells or only a specific cell subset has these capacities is currently unknown. In the current study, we found that Cluster 7 had among its marker genes, some that could be catalogued as innate genes, commonly found in DCs or macrophages. Hence, two genes encoding orthologues of CD83 were identified in this subpopulation as marker genes. Interestingly, CD83 has been previously associated to a population of small mononuclear blood cells from Atlantic salmon which also expressed high MHC-II levels, showed a high phagocytic capacity and the ability to differentiate into DC-like cells [32]. However, in Atlantic salmon, no B cell markers were identified in this subpopulation, whereas in our study, the transcription of different immunoglobulins and CD79 unequivocally indicates that these cells correspond to a B cell subset. Other important markers that link this B cell subpopulation with innate responses are the genes annotated in the rainbow trout genome as CCL4 and *tnfrsf9* (CD137), both known to play important roles in monocyte activation. Interestingly, a significant increase in the levels of transcription of both of these genes had also been reported in rainbow trout IgM^+^ B cells after stimulation with either T-dependent or T-independent antigens [33]. Rainbow trout CCL4 was originally identified as a highly inducible chemokine produced by differentiated rainbow trout macrophages after LPS induction [34]. However, it is important to mention that after this first description, posterior studies focused at establishing phylogenic relationships between mammalian and fish CC chemokines renamed this gene as CK5B [35] and established a close relation with mammalian CCL5 [35,36]. In mammals, CCL5 is known to attract T cells, specifically CD4^+^ Th1 cells [37]. In rainbow trout, a previous study established that upon viral infection, CpG or poly I:C stimulation, IgM^+^ B cells increased CK5B transcription. This was interpreted at the time as a mechanism of B cells to attract and obtain co-stimulatory signals from T helper cells [38]. Therefore, it might be possible that this is the case for this B cell subset identified.

Cells from cluster 7 also showed a differential expression of several transcription factors such as *irf4* and *irf8* associated in mammals with different functions throughout the B cell differentiation process. For instance, the combined loss of IRF4 and IRF8 in mammals resulted in a blockage of germline κ and λ gene transcription as well as DNA recombination at the pre-B cell stage [39]. In addition, IRF4 is a common marker for activated B cells known to promote Ig CSR as well as plasma cell differentiation by positive regulation of the *Aicda* and *Blimp1* genes, respectively [40]. Other transcription factors identified as markers for this cluster also point these cells having experienced some kind of activation by either an antigen or another type of stimuli. For instance, an increased transcription of different Egr-1 homologues had also been observed in mature mammalian B cells which undergo proliferation as a consequence of B cell receptor (BCR) cross-linking [41]. The participation of this transcription factor in the differentiation program of B cells into plasma cells has also been evidenced [42]. Egr-3, on the other hand, has been identified as an activator of suppressor of cytokine signaling-1 (SOCS1) and SOCS3, inhibitors of STAT1 and STAT3, which regulate B cell function in adaptive immune responses and homeostasis by promoting antigen receptor signaling [43]. Fra-2, also identified as a key transcription factor in this cluster, is involved in the proliferation and differentiation of B cells acting as an upstream regulator of Foxo1 and Irf4 expression [44]. Thus, all the marker genes identified in the complex regulatory network identified among the markers of cluster 7 seem to indicate that these CD83^+^ cells characterized by the expression of genes commonly related with macrophages experienced activation. 

### 4.3. Cells in Cluster 1 Correspond to IgT+ B Cells 

Cluster 1 corresponds to approximately 85% of cells with a confirmed IgT transcriptional profile. As previously established, these cells constitute an independent B cell linage with no IgM or IgD [29]. Interestingly, the functionalities and marker genes that were associated with this cluster seemed to indicate an initial stage of maturation. For instance, a significantly higher expression of two genes encoding GATA-binding factors 2-like were found in this cell subset when compared to others, and these factors have been commonly related with primitive hematopoiesis in mammals [45]. In mammals, this transcription factor is expressed in hematopoietic progenitor cells (HPCs) and is considered a key component of the transcriptional program required for early hematopoietic development [45,46]. Similarly, the identification as markers of two genes encoding the pre-B cell leukemia transcription factor 1 (*xbp1*), also support that these cells are at an initial stage of maturation, as this is one of the earliest-acting transcription factors that regulates de novo B-lineage lymphopoiesis, with critical functions during the intermediate stage between hematopoietic stem cell development and B cell commitment [47]. As IgT^+^ cells are known to preferentially colonize mucosal surfaces [48], it might be possible that these cells are still in an immature stage in peripheral blood thus completing their maturation in mucosal tissues. 

Cluster 1 also included several HSPs among its most significant markers. In mammals, the interaction of HSPs with antigen presenting cells (APCs) such as macrophages or DCs, through receptors involved in the innate immune response, such as CD40 or Toll-like receptors 2 and 4 (TLR2/4), leads to several non-antigen specific reactions that promote the stimulation of the innate immune system [49,50]. Thus, the strong overrepresentation of these genes in this cluster may indicate that HSPs play a specific role in the maturation and/or differentiation of IgT^+^ B cells. Remarkably, several innate receptors such as TLR12 and TLR13 were also identified as markers for this subpopulation, being these the only TLRs identified with significant differential expression between cell clusters. Although no homologues have been identified for these genes in humans, both present homologues in mice. Murine TLR12 and TLR13 are known to activate innate immune responses via MYD88 and TRAF6, leading to NF-kappa-B activation, cytokine secretion and an inflammatory response [51,52]. Thus, the expression of these TLRs in IgT^+^ B cells commonly related with mucosal responses [48], suggests that these cells are traveling from central immune organs to mucosal peripheral tissues where they might be activated through the action of these TLRs. 

Several cells within this cluster showed a significant higher expression of two interleukin receptors, *il3r2* and *il31r*. Additionally, some cells from this cluster produced different cytokines that might have effects on other immune cells such as *il15* or *tnfsf13b* (BAFF). Studies in mice have determined that IL-15 together with recombinant CD40 ligand is able to potently induce polyclonal IgM, IgG1, and IgA secretion [53] and together with CpG oligonucleotides to induce the proliferation of class switched human memory B cells [54]. In the case of BAFF, this cytokine is a key factor for B cell survival and maturation, illustrated by the absence of mature B cells in BAFF-deficient mice [55]. Previous work performed in rainbow trout showed the regulation of splenic B cell functionality by BAFF [7] and differential effects on peritoneal IgM^+^ B cell subpopulations [6]. Furthermore, these studies revealed the production of BAFF by a subset of splenic IgM^+^ B cells pointing to an auto-regulatory mechanism of B cell survival in this organ [6]. In this case, the expression of these molecules in peripheral blood IgT^+^ B cells may indicate the capacity of these cells to regulate other B cell subsets or themselves in an autocrine fashion. 

### 4.4. CXCR4-Like 4 Genes as Key Markers for Fish B Cells

Mammalian CXCR4 has been suggested as a marker for an initial common lymphoid progenitor in mice [56] and for differentiated plasmablasts or plasma cells both in mice and humans [57,58]. Several studies have analyzed the evolution and function of *cxcr4* genes in teleost fish showing the presence of either two paralog genes designated as *cxcr4a and cxcr4b* [59,60,61] or even four gene copies in cyprinids [62]. Our results identified four different genes annotated as rainbow trout CXCR4-like, which all seemed expressed in B cells. Interestingly, all of them were identified as potential markers for different B cell subpopulations. On one hand, two genes encoding CXCR4 (LOC110520024 and LOC110501543), which seem to be homologues to the molecule designated as CXCR4b in previous studies, were identified as shared markers for cluster 0 and cluster 8. On the other hand, the other two genes encoding homologues of the molecule previously designated as CXCR4a (LOC110516585 and LOC110530627) showed differential expression in cluster 1 and cluster 5, respectively. A previous study in fish pointed to the duplication of CXCR4 genes in teleosts evolving to a gene subfunctionalization that may stabilize the immune system controlling teleost hematopoietic stem/progenitor cell (HSPC) homeostasis [61]. In addition, these authors suggested that HSPCs expressing CXCR4a preferentially bind LPS, whereas those expressing CXCR4b preferentially bind SDF-1 reflecting different pathways of immune cell differentiation [61]. Our analysis focused in B cells seems to indicate that CXCR4 relates to an advanced state of maturation, but with different rainbow trout CXCR4 molecules carrying out a differential regulation of specific B cell subpopulations. 

### 4.5. Long Non-Coding RNAs Seem to Regulate Different B Cell Transitional Stages in Fish

LncRNAs carry out an epigenetic regulation through chromatin modification of differentiating enhancer-associated lncRNAs (eRNAs), acting in cis, and promoter-associated lncRNAs (pRNAs), acting in trans [63]. The potential regulatory function of lncRNAs during B cell differentiation and development has recently emerged [64]. In humans, a set of over 3000 lncRNAs were detected analyzing bone marrow and thymic progenitors spanning the earliest stages of B and T lymphoid specification [65]. The expression patterns observed along these lncRNAs identified were shown to be highly stage-specific and more lineage-specific than protein-coding genes [65]. More recently, RNAseq or microarray studies using isolated B cell subpopulations highlighted equivalent expression patterns between coding genes and lncRNAs throughout B cell differentiation [66,67,68]. Similarly, in mouse, a repertoire of 4516 lncRNAs were identified with expression along 11 mouse B cell subpopulations, again suggesting that lncRNAs displayed a greater cell-type restriction than coding genes, showing very restricted spatiotemporal roles during B cell development [67]. Interestingly, the expression of lncRNA loci is controlled by transcription factors specific of B cell lineage, such as for example PAX5, known to regulate several lncRNAs in both pro-B and mature B cells [67].

Our results identified 60 transcripts, annotated in the *O. mykiss* genome as lncRNAs, which showed significant differences in expression levels between cell clusters. Thus, four clusters had a lncRNA as its most significant marker, in agreement with the cell-type restriction previously described in mammals. 

Interestingly seven lncRNAs were identified as markers for cluster 3 in correlation with a significant enrichment in genes related with “regulation of gene expression, epigenetic”. In addition, several functions showing significant enrichment in this cluster were related to the modification or assembly of different cellular organelles. For instance, the *pink1* gene was identified among the most significant markers encoding a protein in this cluster. In mammals, PINK1 modulates mitochondrial trafficking and is involved in the clearance of damaged mitochondria via selective mitophagy [69]. Recent studies in mammals have evidenced important links between mitophagy and immunity [70,71,72]. Mitochondrial stress in response to pathogens and danger signals induces mitophagy through the PINK1/Parkin pathway [73]. Interestingly, an important role has been attributed to PINK1 in integrating extracellular signals with the metabolic state, thus determining T cell fate [74]. Although there are no evidence in mammals that connect PINK1 with B cell fate, our results suggest a potential connection in fish defining a specific subpopulation of peripheral blood B cells from rainbow trout. 

Cluster 4 also has a high number of lncRNAs among their most significant markers. In this cluster, several enzymes related to different mechanisms of glycosylation were also identified as markers. Both N- and O- glycosylations have emerged as important translational modifications of key immune proteins including Igs [75,76,77]. These modifications have also been observed in the variable regions of these Igs, affecting both antibody specificity and stability [78,79]. In human blood B cells, IgG1 Fc-glycosylation with all-trans retinoic acid has been described upon stimulation [80]. Hence, our results might indicate that B cells in Cluster 4 have already encountered some kind of stimuli that has triggered a glycosylation process.

In what concerns lncRNAs, it should be mentioned that cells from cluster 5, which seemed associated with CXCR4a expression, were also highly represented by the expression of the lncRNA LOC110521442. Finally, cluster 9 is also highly represented by the lncRNA LOC110513619. Interestingly, a significant enrichment in functions related with fatty acid oxidation was observed in this cluster reflecting a specific metabolic state of this B cell subpopulation. Recent results highlighted fatty acid oxidation as a predominant energy source for *ex vivo bona fide* GC B cell growth [81]. Although no GCs have been described in fish, our results suggest that fatty acid metabolism may represent an important function in specific B cell subpopulations, opening a new field to further explore B cell immunometabolism in fish. 

Altogether, our results suggest for the first time that fish lncRNAs also carry out key roles in B cell functionality, showing a very restricted spatiotemporal expression. Although the specific functions carried out by these regulators remain to be further analyzed, the correlation with B cell subpopulations associated to important functions suggests a role of lncRNAs in maintaining these different B cell states. 

## 5. Conclusions

In summary, we have taken advantage of the recently developed 10× Genomics single cell sequencing techniques to analyze the transcriptome of 3984 MHC II^+^ lymphoid cells from rainbow trout peripheral blood. These cells, obtained from three independent fish, mostly corresponded to B cells as established by Ig transcription levels or expression of the B cell marker CD79. This methodology allowed us to establish 10 different B cell clusters, characterized by a specific gene expression profile. These fish were obtained from a fish farm as adults, thus throughout their development they might have encountered different stimuli, microorganisms or even pathogenic agents. For this reason, the B cell population analyzed will surely include cells in different stages of activation. However, whether the different cell clusters identified correspond to cells in different stages of activation/differentiation or in fact are diverse naïve B cell subpopulations that carry out different immune functions should be further explored. In any case, our studies highlight the diversity of the B cell response in teleost and provide further insight on key genes that mediate important processes throughout the B cell maturation/differentiation process in these species, also providing us with a panel of potential markers that might be used in the future to differentiate and further explore the functionality of these B cell subsets.

## Data Availability

The data discussed in this publication have been deposited in NCBI’s Gene Expression Omnibus [82] and are accessible through GEO Series accession number GSE158102 (https://www.ncbi.nlm.nih.gov/geo/query/acc.cgi?acc=GSE158102; Public since 10 May 2021).

## Supplementary Materials

The following are available online at https://www.mdpi.com/article/10.3390/biology10060511/s1, Table S1. Results from marker identification analysis. Rainbow trout genes showing significant differential expression (adjusted *p* < 0.001) between cell clusters (identified with different colors). Table S2. Results from GO term single enrichment analysis. GO terms significantly enriched (adjusted *p* < 0.05) within markers from each cell cluster for each Gene Ontology category (Biological process, Molecular function and Cellular component). Different clusters are represented by different colors.
